# *Rhynchotermes armatus*, a new mandibulate nasute termite (Isoptera, Termitidae, Syntermitinae) from Colombia

**DOI:** 10.3897/zookeys.892.38743

**Published:** 2019-11-27

**Authors:** Rudolf H. Scheffrahn

**Affiliations:** 1 Fort Lauderdale Research and Education Center, Institute for Food and Agricultural Sciences, 3205 College Avenue, Davie, Florida 33314, USA Institute for Food and Agricultural Sciences Davie United States of America

**Keywords:** endemic, Magdalena Valley, taxonomy, vicariant divergence

## Abstract

*Rhynchotermes armatus***sp. nov.** is described from soldiers and workers collected in the Magdalena River Valley of Colombia. Both castes of this new termite are superficially similar to *R. perarmatus* (Snyder) but the former are smaller, head capsules yellowish instead of reddish, and among additional characters, the soldier has narrower mandibles and marginal teeth.

## Introduction

The genus *Rhynchotermes* Holmgren constitutes a peculiar group of neotropical termites ranging from Belize to Argentina. They feed openly on surface litter during crepuscular or nocturnal forays. They nest underground or in shallow epigeal nests. Soldiers are either monomorphic or weakly dimorphic and workers are monomorphic. A thorough revision of *Rhynchotermes* by Constantini and Cancello (2016) included seven species: *R. amazonensis* Constantini & Cancello, *R. bulbinasus* Scheffrahn, *R. diphyes* Mathews, *R. matraga* Constantini & Cancello, *R. nasutissimus* (Silvestri), *R. perarmatus* (Snyder), and *R. piauy* Cancello. I herein describe an eight species, *R. armatus* sp. nov., from Colombia.

## Material and methods

Live specimens were preserved in 85% ethanol. External and internal worker morphology was depicted using two different methods. In the first method, soldiers and workers were suspended in Purell Instant Hand Sanitizer in a plastic Petri dish. This allowed for transparent posturing and support during photography using a Leica M205C stereomicroscope controlled by Leica Application Suite version 4.0 montage software. The worker enteric valve armature (**EVA**) was prepared for photography by removing the entire worker P2 section in ethanol. Food particles were expelled from the P2 tube by pressure manipulation. The tube was quickly submerged in a droplet of PVA medium (BioQuip Products Inc.) which eased muscle detachment by further manipulation. The remaining EVA cuticle was longitudinally cut, splayed open, and mounted on a microscope slide using the PVA medium. The EVA was photographed with a Leica CTR 5500 compound microscope with bright field optics using the same montage software.

## Taxonomy

### Rhynchotermes armatus

Taxon classificationAnimaliaBlattodeaTermitidae

Scheffrahn sp. nov.

DAFE6CCF-7F2A-5DA0-91C9-2B4FFF95BC87

http://zoobank.org/6BAF94E6-EA51-4A1B-B300-92151D9F98F4

Figs 1 , 2 , 3

Rhynchotermes perarmatus : Pinzón and Castro 2018 [Colombia, Huila, El Agrado].

#### Material examined.

**Figure 1.**
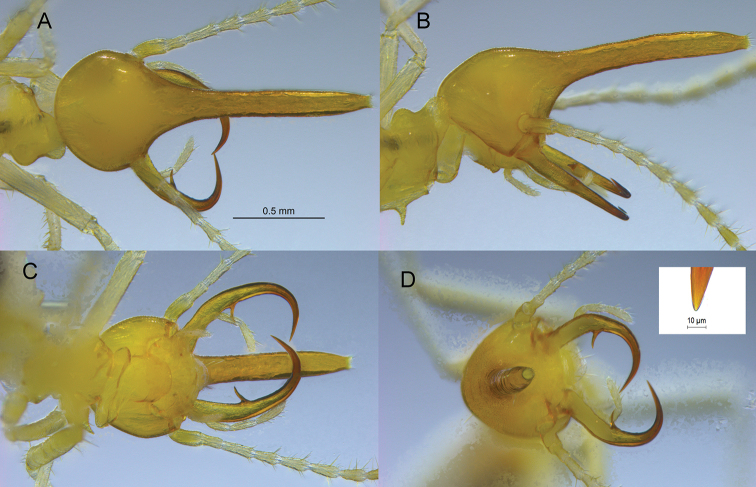
*Rhynchotermes armatus* sp. nov. soldier **A** dorsal **B** lateral **C** ventral and **D** anterior views (inset is tip of left apical tooth).

Colombia: Pandi, Depto. Cundinamarca (4.13, -74.49; Elev. 930 m), 23JAN96, col. J. Krecek. Three colonies: CO879, **holotype** soldier (Fig. 1), one other soldier, 30 workers, and three larvae; CO880, eight soldiers and six workers; CO881, 17 soldiers and two workers (Fig. 3). All material is housed at the University of Florida Termite Collection in Davie, Florida, USA.

#### Description.

The revised description of the genus *Rhynchotermes* by Constantini and Cancello (2016) includes all characters found in *R. armatus*.

#### Imago.

Unknown.

***Soldier*** (Figs 1, 3; Table 1). Monomorphic. Head capsule, nasus, and mandible bases straw yellow; apical and marginal teeth yellow-brown. Head capsule dome shaped in lateral view. Nasus projecting well beyond mandibles; nearly cylindrical, tapering to rather large circular opening. Nasus hollow, thickness of outer wall even. Pilosity of head capsule limited to two faint setae near level of antennal fossae and one or two even shorter and fainter setae on vertex. Nasus without setae; curved slightly downward. Mandibles curved ~150–180° with greatest curvature beyond marginal teeth. Mandibles narrowing beyond marginal teeth. Apical teeth exceptionally narrow and sharp; marginal teeth extremely thin and angled ~60° toward labrum. Antennae very long, about twice the length of the nasus; 14 articles, 2<3>4=5. Pronotum slightly lighter than head; asymmetrically bilobed in dorsal view, posterior lobe larger; in lateral view, margin of anterior lobe continuous with vertex, posterior lobe forming hump. Fore coxae with thorn-like process, slight downward curvature.

**Table 1.** Measurements (mm, *N* = 12) of *Rhynchotermes armatus* sp. nov. soldiers from three colonies.

**Measurements**	**Max**	**Min**	**Mean**
Head length with nasus	2.20	1.73	2.06
Head max. width	0.88	0.67	0.81
Pronotum width	0.56	0.37	0.52
Length of hind tibia	1.54	1.19	1.39
Max. length L mandible	1.05	0.61	0.89
No. antennal articles	14	14	14

**Figure 2.**
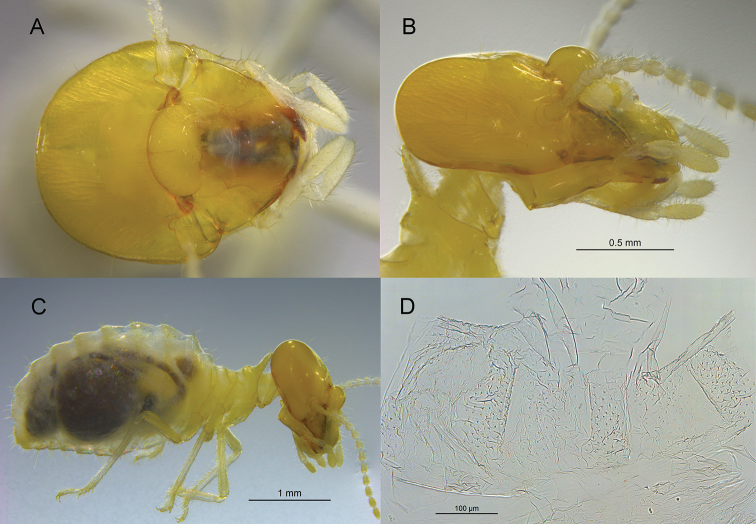
*Rhynchotermes armatus* sp. nov. worker **A** dorsal and **B** lateral views of head capsule **C** lateral view of habitus and **D** enteric valve armature cut longitudinally and laid flat.

***Worker*** (Fig. 2; Table 2). Monomorphic. Head capsule concolorous with soldier. Head capsule with eight-to-ten long, evenly spaced setae; postclypeus strongly inflated. In lateral view, posterior lobe of pronotum much longer and angled ca. 130° from plane of posterior lobe. Antennae with 14 articles. Forecoxa with elevated rise on anterior margin. Enteric valve weakly armed consisting of three rectangular cushions, each with 30–40 very small triangulate spines; cushions separated by wider cuticular lining interspersed with even smaller spines.

**Table 2.** Measurements (mm, *N* =12) of *Rhynchotermes armatus* sp. nov. workers from three colonies.

**Measurements**	**Max**	**Min**	**Mean**
Head length to condyles	1.00	0.61	0.79
Head width	1.25	1.09	1.17
Pronotum width	0.70	0.58	0.63
Length of hind tibia	1.37	1.16	1.26
No. antennal articles	14	14	14

#### Comparison.

Constantini and Cancello (2016) divided *Rhynchotermes* soldiers into two morphogroups: those with mandibles larger than the head (*R. perarmatus* and *R. bulbinasus*) and those with mandibles shorter than the head (all remaining species). *Rhynchotermes armatus* falls into the former group and is closest *R. perarmatus*, each having a tubular nasus and large mandibles. Snyder (1925a) reported a soldier head length of 2.5–2.6 mm for *R. perarmatus* and 3.0–3.2 mm for its junior synonym, *R. major* (Snyder 1925b) from Panama and Costa Rica, respectively. These measurements are from 1.14 to 1.85 times larger than *R. armatus*.

**Figure 3.**
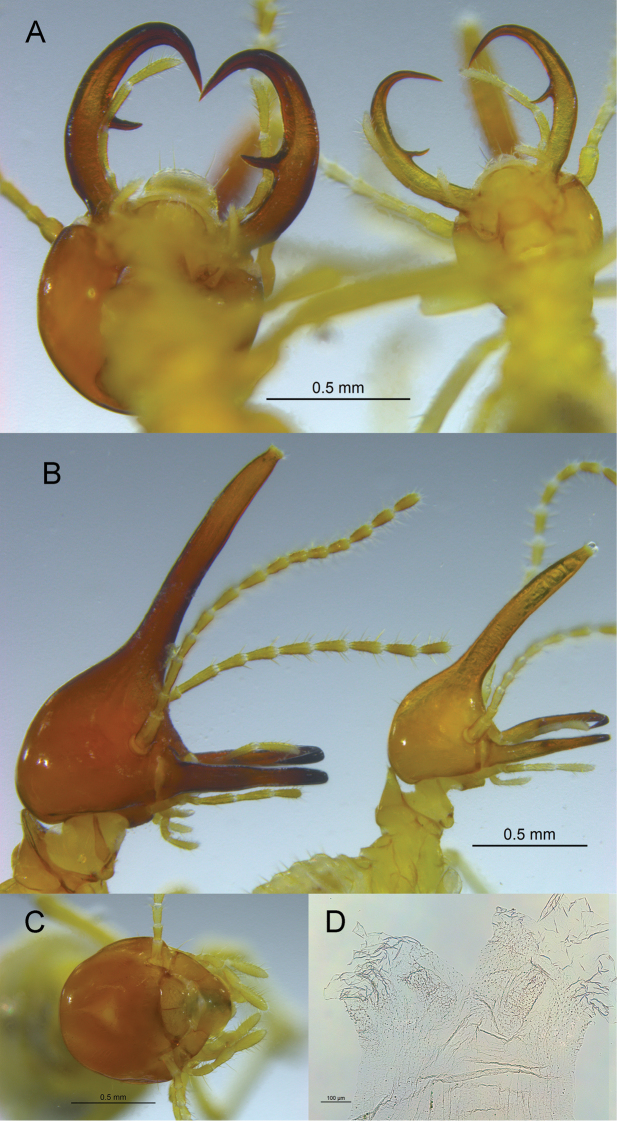
**A** Ventral view of soldiers of *Rhynchotermes perarmatus* from Panama (left PN153) and *R. armatus* sp. nov. from Colombia (right, CO881) **B** same as **A**, but lateral view **C***R. perarmatus* worker and **D** worker enteric valve armature (PN153).

In addition to the head length, the *R. armatus* soldier is smaller in all measurements, has a yellowish head pigmentation versus reddish, and has narrower (thinner) mandibles, including the marginal teeth (Fig. 3A). The nasus of *R. armatus* has a greater curvature and the third antennal article is proportionately shorter (Fig. 3B). The workers of both species are concolorous with their respective soldiers. The *R. armatus* worker has a small and faint fontanelle (Fig. 2A) compared to the proportionately larger and more contrasting fontanelle and cranial suture of *R. perarmatus* (Fig. 3C). The *R. armatus* worker EVA has fewer spines on the three cushions and the inter-cushion areas than *R. perarmatus*. Also the *R. perarmatus*EVA has longitudinal folds covered with fine fringes anterior to the cushions (Fig. 3D) which are lacking in *R. armatus*.

The next closest species, *R. bulbinasus*, differs from the other two in having a median inflation of the nasus. The apical teeth of *R. armatus* are extremely sharp (point ca. 1.5 µm wide, Fig. 1D inset), probably only comparable with those of *Silvestritermes gnomus* (Constantino) (https://www.termitediversity.org/sandsi-sinocapri?lightbox=dataItem-jy00jfyy).

#### Etymology.

The species name is a truncated derivative of its closest congener, *R. perarmatus*. Although Snyder (1925a) did not provide an etymology for *R. perarmatus*, he gave an apt analogy for the gestalt of all *Rhynchotermes* soldiers: “It is a thoroughly armed species and runs about audaciously with its nasus or beak elevated at an angle of 45°, reminding one of an antiaircraft gun”.

### Key addition

A new couplet (3) for the *Rhynchotermes* key by Constantini and Cancello (2016) is offered below to accommodate *R. armatus*:

**Table:** 

1	Mandibles larger than head, clearly visible from dorsal view when closed; apical region of each mandible extending well beyond the opposite mandible when closed (fig. 6B)	**2**
–	Mandibles shorter than head capsule, barely or not visible from dorsal view when closed; apical region of each mandible aligns to the proximal region of the opposite mandible when closed (fig. 6A)	**4** ^1^
2	Proximal region of frontal tube constricted; apical region bulbous (figs 3C, 4C)	***R. bulbinasus***
–	Frontal tube elongate, subcylindrical (figs 3H, 4H)	**3**
3	Length of head with nasus ≤ 2.20 mm, head yellowish	***R. armatus***
–	Length of head with nasus ≥ 2.5 mm, head reddish brown	***R. perarmatus***

## Discussion

The long-mandible clade (*R. perarmatus*, *R. bulbinasus*, and *R. armatus*) are restricted to Central America and northern Colombia (localities herein; Constantini and Cancello 2016) with one unconfirmed report of *R. perarmatus* from Ecuador (Snyder 1949). All remaining *Rhynchotermes* species have an Amazonian or austral distribution (Constantini and Cancello 2016).

The Magdalena River Valley lies between the central and eastern ranges of the Colombian Andes and is host to various endemic faunas including birds (Cracraft 1985), fishes (Anderson and Maldonado‐Ocampo 2011), frogs (Ospina-Sarria et al. 2015), lizards (Velasco and Hurtado-Gómez 2014), and insects (Huertas et al. 2009; Padilla-Gil 2015). *Rhynchotermes armatus* is apparently another endemic species from the Magdalena River Valley. It is plausible that *R. armatus* and *R. bulbinasus* evolved from an ancestor of *R. perarmatus* following the gradual land bridge closure joining Central and South America during the Miocene (Woodburne 2010). This timeframe coincides with the orogenic rise of the Eastern Cordillera of Colombia (Gregory-Wodzicki 2000) which may have led to the vicariant divergence and allopatric speciation across elevational gradients leading to one or both Colombian species. This scenario was reported for *Rheobates* frogs of the same region (Muñoz Ortiz et al. 2015). Additional undescribed termites known only from this valley include a new genus of Apicotermitinae, a new species of *Rugitermes*, and a new *Obtusitermes* (Scheffrahn and Pinzón, unpublished). No doubt further exploration will yield more new taxa.

## Supplementary Material

XML Treatment for Rhynchotermes armatus

## Footnotes

Couplet 4 replaces 3 in original key so a digit is added to all ensuing couplets.
